# Supplementary figures and images for: Enhancing intrusion detection performance using explainable ensemble deep learning
Source: PeerJ Comput Sci. 2024 Sep 13;10:e2289. doi: 10.7717/peerj-cs.2289 (PMC11419647; doi:10.7717/peerj-cs.2289)

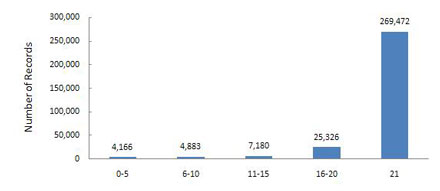

Supplement: Supplemental Information 2 [file peerj-cs-10-2289-s002.zip › KDDTest1.jpg]

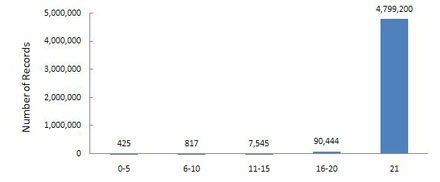

Supplement: Supplemental Information 2 [file peerj-cs-10-2289-s002.zip › KDDTrain1.jpg]
